# Review of electroencephalography and electromyography research in robotics: opportunities and challenges

**DOI:** 10.1186/s42492-026-00216-2

**Published:** 2026-03-20

**Authors:** Zefeng Wang, Meiyan Xu, Junfeng Yao, Yue Yu, Bingbing Hu, Yufei Wang, Yu Wang, Xiaopeng Zhang

**Affiliations:** 1 School of Informatics, Xiamen University, Xiamen, 361102 Fujian China; 2 School of Film, Xiamen University, Xiamen, 361005 Fujian China; 3 Key Laboratory of Digital Protection and Intelligent Processing of Intangible Cultural Heritage of Fujian and Taiwan Ministry of Culture and Tourism, Xiamen, 361005 Fujian China; 4 School of Computer Science, Minnan Normal University, Zhangzhou, 363000 Fujian China; 5 School of Medicine, Dongfang Hospital of Xiamen University, Xiamen University, Xiamen, 361102 Fujian China; 6 Institute of Automation, Chinese Academy of Sciences, Beijing, 100190 China

**Keywords:** Electroencephalography, Electromyography, Neuroscience, Robotics, Human-machine interface, Data acquisition, Neuromuscular intentions

## Abstract

In the evolving nexus of neuroscience and robotics, the symbiotic fusion of electroencephalography (EEG) and electromyography (EMG) is emerging as a paradigm-shifting avenue for enhancing human-machine interfaces. While EEG, which captures the subtle electrical nuances of the brain, offers a potent channel for nuanced brain-machine communication, EMG serves as a bridge, converting neuromuscular intentions into actionable directives for robotic apparatuses. This review highlights the current methodologies in which EEG and EMG not only function in silos but also converge harmoniously to dictate robotic control. By delving deeper into this, the intricate synergy between cognitive processes, muscular responses, and machine actions can be unraveled. Subsequently, the discourse also navigates through the myriad challenges encountered in realizing real-time, seamless integration of these bio-signals with robotics and the innovative solutions poised to address them. The aim is to provide a comprehensive understanding of the interplay between neuroscience and robotics. This insight will help drive breakthroughs in adaptive human-machine collaboration.

## Introduction

Robotics has made significant progress as a multidisciplinary field by integrating advances in computer science, mechanical engineering, and neurobiology. Critical developments include the emergence of reinforcement learning techniques that allow robots to adapt dynamically to ever-evolving environments, inspiration derived from nature leading to biomimetic robotic designs [1–3], and the exploration of learning human motions that can better serve the needs of humans [4–6]. In addition, the integration of robots into everyday human environments underscores the significance of human-robot collaboration [7], necessitating innovations in robot perception and cognition [8]. Robotics is also important for human exoskeletons [9–11] and rehabilitation [12, 13]. Simultaneously, with the proliferation of social and multiple robots, human-robot interactions (HRIs) have become enriched [14–16]. However, the translation of biological signals such as electroencephalography (EEG) and electromyography (EMG) into robotic commands is of considerable relevance [17], providing a bridge between the realms of biological computation and mechanical autonomy.

**Fig. 1:**
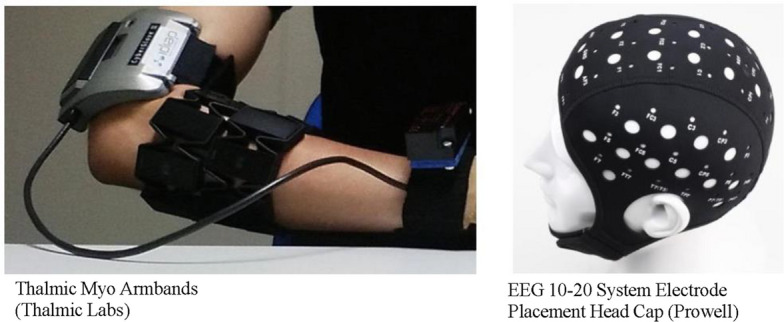
Electromyography [19] and electroencephalography [20] devices

EEG and EMG are noninvasive techniques for recording the electrical activity of the brain and skeletal muscles, respectively [18]. The signals are collected using different devices (Fig. 1). EEG signals, which represent the collective behavior of neurons in the brain, have numerous applications, particularly in the control of robotic systems [21]. EMG, on the other hand, captures the electrical potentials generated during muscle contractions [22], providing information about muscular activity and, by extension, bodily movements [23]. Although both modalities offer unique insights, their potential synergy is a promising area for exploration [24].

EEG and EMG signals are instrumental in numerous areas of robotics. In HRI, these biological signals allow intuitive control and communication [25, 26] and provide a direct interface for amputees to control robotic limbs [27–29].

Despite significant progress, the implementation of EEG and EMG in robotics presents technical and scientific challenges. These problems include noise interference, signal variability, and computational complexity. Current research efforts are directed toward addressing these challenges and paving the way for robust, reliable, and practical bio-signal controlled robots [30].

**Fig. 2:**
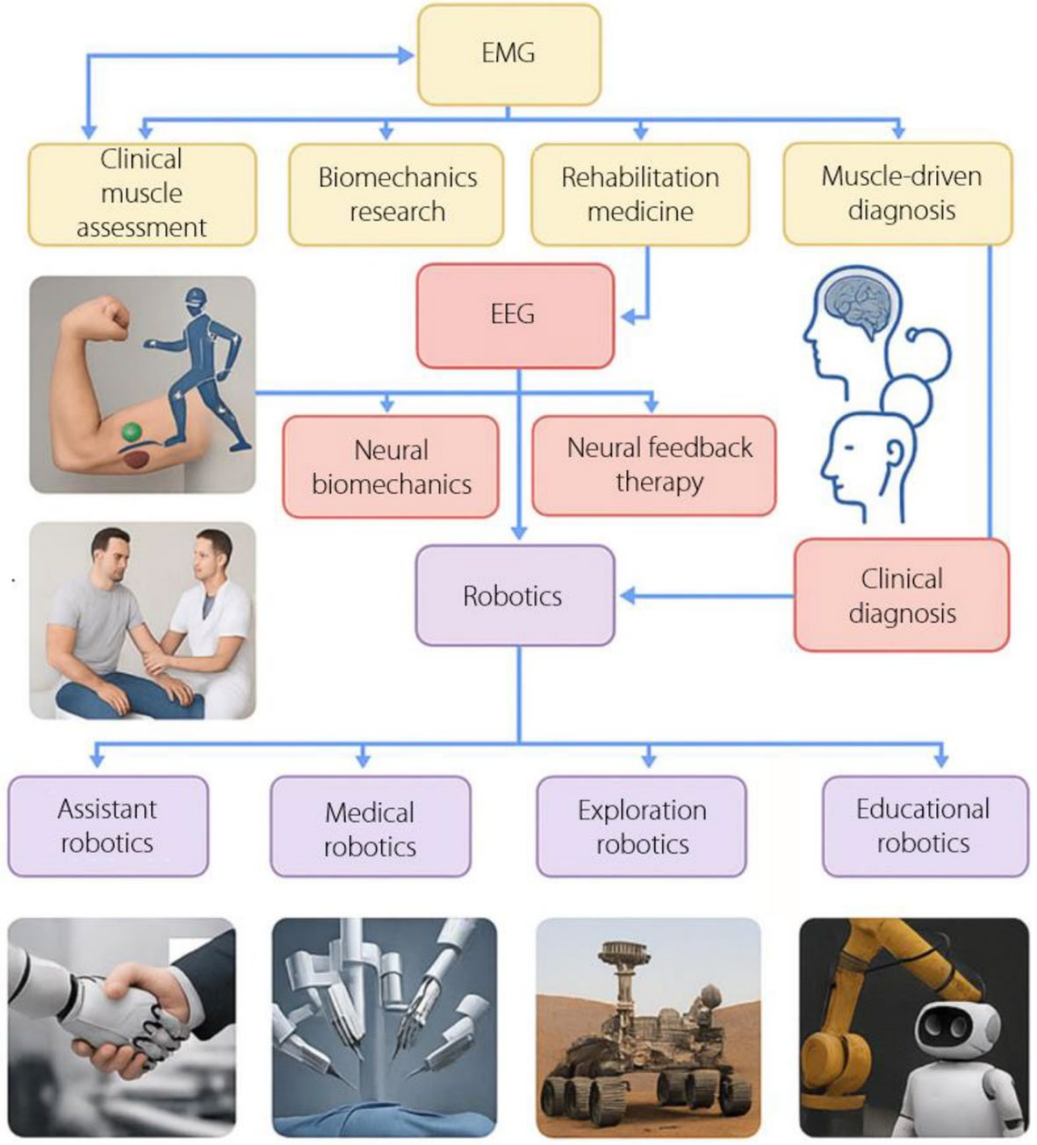
Relationship between electroencephalography, electromyography and robotics. EEG: Electroencephalography; EMG: Electromyography

Numerous studies have explored the integration of EEG and EMG signals with robotic systems, demonstrating their potential for enhancing various aspects of robotics. For instance, Shao et al. [31] demonstrated the use of EEG for mental workload assessment in robot operators, enabling adaptive control mechanisms to optimize human-robot collaboration. In contrast, Nadjib et al. [32] investigated the application of EMG signals for hand gesture recognition, facilitating the intuitive and dexterous control of robotic prosthetics. This study aims to provide an in-depth analysis of the advancements, challenges, and future opportunities in utilizing EEG and EMG signals to enhance the capabilities and interactions of robotic systems. Figure 2 shows the relationship between EEG, EMG, and robotics. The diagram delineates the significance of EEG in clinical diagnosis, neurofeedback therapy, brain-machine interface (BMI) development, and neurobiomechanics research. In the realm of EMG, its pivotal roles in clinical muscular assessment, biomechanics investigations, muscle-driven robotics, medical procedures [33], and rehabilitation medicine have been highlighted. Robotics, as a distinct domain, is further subdivided to highlight its applications in assistive, medical, exploratory, industrial, and educational contexts. Notably, BMI has emerged as an integrative nexus, bridging EEG, EMG, and robotics, and underscoring the interdisciplinary synergy of these fields.

Beyond EEG and EMG, modern human-robot systems can also leverage eye tracking and electro-oculography for pointing and selection, hemodynamic and autonomic signals (e.g., functional near-infrared spectroscopy (fNIRS), electrocardiography, electrodermal activity) for state monitoring, and multiple EEG paradigms–motor imagery (MI), steady-state visually evoked potentials (SSVEPs), P300 (P300 event-related potential)/rapid serial visual presentation (RSVP)–that can all generate machine-interpretable commands. This work acknowledges this broader landscape and provides brief pointers where relevant. However, this review is intentionally robotics-centric and focuses on EEG + EMG as a coupled interface because the pair uniquely matches robotics’ control contracts. Specifically, EMG affords low latency, continuous modulation of forces/impedances, and joint-level behaviors, while EEG is the most reliable for sparse, high-level intent such as mode switching or goal confirmation under longer latencies and lower SNR. This complementarity enables principled intent-autonomy arbitration and safety-constrained fusion inside robot controllers–capabilities that gaze/electro-oculography excel to augment for target selection but do not replace for continuous torque/velocity scaling, and that fNIRS or autonomic measures typically cannot meet at robotic time scales. Therefore, the motivation is not to exhaust all biosignals or paradigms *per se* but to answer a robotics question: when and how EEG-EMG coupling measurably improves closed-loop robot performance (task success, safety envelopes, operator workload, and adaptation time) compared with single-modality or non-myoneural alternatives. Accordingly, this study (1) positions MI/SSVEP/P300/RSVP as encoding strategies within our taxonomy, discussed insofar as they interface with robotic control; (2) treats EMG feature learning and synergy mapping as the continuous control backbone; and (3) analyzes the decoding/encoding codesign with embodiment and shared autonomy while briefly contrasting auxiliary modalities in context. This scope clarifies why the title emphasizes EEG and EMG, yet situates them among–and not apart from–the wider biosignal family, aligning this review with the constraints and evidence that matter for deployable robots.

**Fig. 3:**
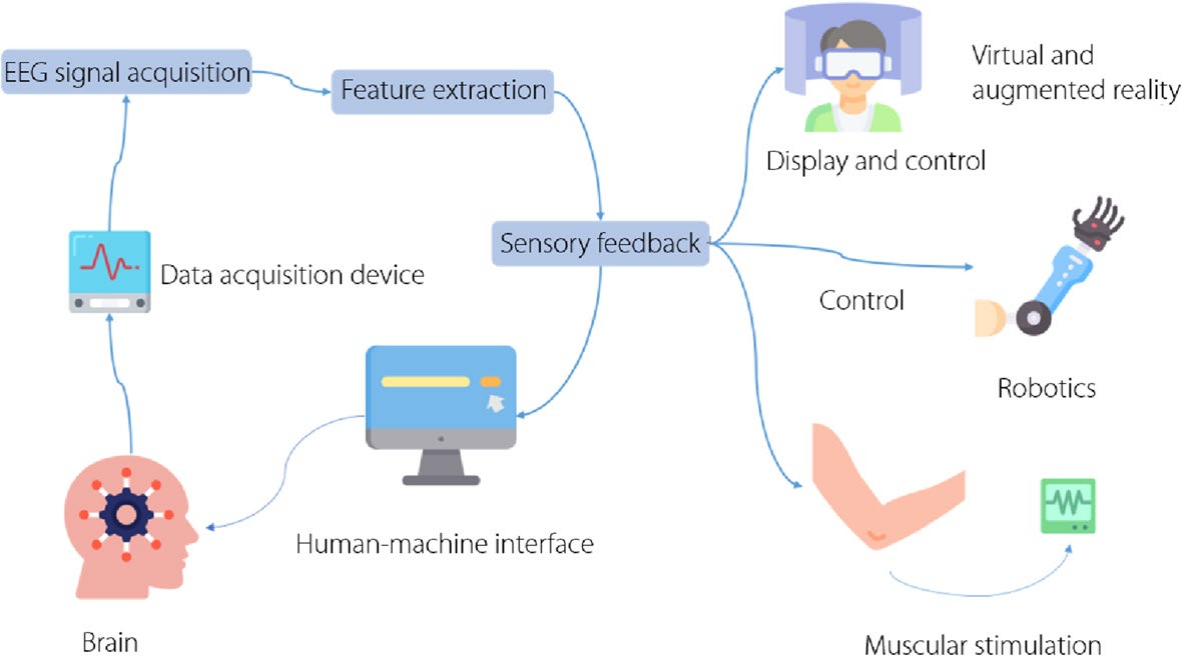
This diagram depicts a brain-computer interface system, illustrating the process flow from electroencephalography signal acquisition to the application of sensory feedback, virtual/augmented reality, robotics, and muscular stimulation. EEG: Electroencephalography

By reviewing these and other relevant studies, refs. [25] and [34] provided an overview of the underlying principles and techniques of EEG and EMG in robotics; delved into the challenges associated with signal acquisition, processing, and interpretation; and examined diverse applications in fields such as assistive robotics and rehabilitation (Fig. 3), and human-machine interfaces.

This review encompasses a broad range of topics related to EEG and EMG in the field of robotics. It includes, but is not limited to, the principles and applications of EEG and EMG in robotics, the analysis and interpretation of EEG and EMG signals, the development of algorithms and techniques for extracting meaningful information from these signals, and the integration of EEG and EMG with robotic systems. This review explores the opportunities and limitations of EEG and EMG in robotics, addressing factors such as signal reliability, noise mitigation, and user adaptation. It also highlights the ethical considerations surrounding the use of neural signals in robotics, such as privacy concerns and informed consent.

The subsequent subsections of this paper discuss the principles and techniques of EEG and EMG, their application in robotics, the challenges they present, and the key studies that have shaped this field.

## Principles and techniques of EEG and EMG in robotics

EEG is a noninvasive neuroimaging technique used to record the electrical activity of the brain [35]. Electrodes are placed on the scalp to detect the synchronized neural firing of brain neurons. These electrical signals, known as brainwaves, are categorized into different frequency bands such as alpha (8–13 Hz), beta (13–30 Hz), theta (4–8 Hz), and gamma (> 30 Hz). EEG provides valuable insights into various cognitive and affective states, mental workloads, attention, and motor intentions. EEG-based brain-computer interfaces (BCIs) have garnered significant interest in the field of robotics, such as EEG-based brain-controlled robots, advances in decoding algorithms, and applications of BCIs in various domains including HRI, rehabilitation robotics, and prosthetics [19]. BCIs allow direct communication between the human brain and robotic systems, enabling users to control robotic movements through imagined tasks or MI. By decoding specific patterns in EEG signals, BCIs enable brain-controlled robotic applications such as robot navigation, object manipulation, and rehabilitation devices [25].

The main principle of EEG-based BCIs in robotics is to establish a direct communication pathway between the human brain and robotic systems. By decoding EEG signals associated with specific mental states or MI, BCIs enable users to control robotic devices or seamlessly interact with them through their thoughts.

EEG plays a significant role in robotics, particularly in the development of BCIs for robot control, cognitive load assessment, emotion recognition, and HRIs. These applications demonstrate the potential of EEG in enhancing the naturalness, adaptability, and user-centricity of robotic systems, paving the way for more intuitive and efficient human-robot collaborations in various domains.

EMG is used to record the electrical activity of the skeletal muscles during voluntary or involuntary contractions. Noninvasive EMG imaging has also matured [36]. Surface or intramuscular electrodes capture these signals, which provide valuable information about muscle activation patterns, force generation, and motor control [37]. EMG has shown promising applications in robotics, particularly in the domain of prosthetics and exoskeletons [38]. EMG-based gesture recognition allows the intuitive control of robotic prosthetics, enabling individuals with limb loss to perform natural and dexterous movements [39, 40]. EMG-driven exoskeletons [41] offer assistance and rehabilitation to individuals with mobility impairments, enabling them to regain mobility and improve their quality of life.

### Related key hardware and technologies

Low-latency links bridge wearables to robots: USB CDC or WiFi/UDP for high-throughput, BLE for untethered use, and MCU bridges into deterministic buses (controller area network/controller area network-FD, EtherCAT). Cross-system clocks are aligned via hardware triggers or IEEE-1588/precision time protocol; thus, biosignal timestamps, joint encoders, and vision/inertial measurement unit frames share a common time base exposed through an robot operating system 2 (DDS) interface. The control is hierarchical: fast surface electromyography (sEMG) drives continuous commands (velocity/torque scaling, grasp aperture) with ≲ 10–30 ms end-to-end latency, whereas slower lower-SNR EEG governs discrete intents (mode switching, goal confirmation) while tolerating ≈ 200–500 ms. The safety arbitration layer blends human intent with autonomy using rate limiters, passivity/energy tanks, and model-predictive safety filters.

Noise reduction ensures the fidelity of biosignal acquisition during dynamic robotic interactions. Joint torque, position, and contact sensors introduce electromagnetic and motion artifacts that are mitigated by hardware-level shielding, medical-grade isolation, and adaptive filtering of synchronized biosignals. The battery power, strain-relieved connectors, and biocompatible encapsulation minimize interference and guarantee safety during long-term operation.

The paradigm design defines how feedback and user intention are integrated to close the sensorimotor loop. Multichannel proprioceptive data–joint torque, contact, or ground-reaction forces–are mapped to vibrotactile or functional electrical stimulation (FES) cues to form a bidirectional control paradigm that enhances embodiment and reduces muscle co-contraction. Ergonomic don/doff mechanisms and user-specific calibration protocols support real-time adaptation without interrupting control cycles.

Decoding algorithms leverage online personalization and adaptive normalization to maintain robust control performance under electrode displacement, sweating, and fatigue. Few-shot recalibration and domain adaptation frameworks continuously update decoder parameters without full retraining. These techniques constitute deployable pipelines for prosthetics, exoskeletons, and teleoperation platforms. They rely on reproducible synchronization, hardware-in-the-loop validation, and gated model updates.

**Fig. 4:**
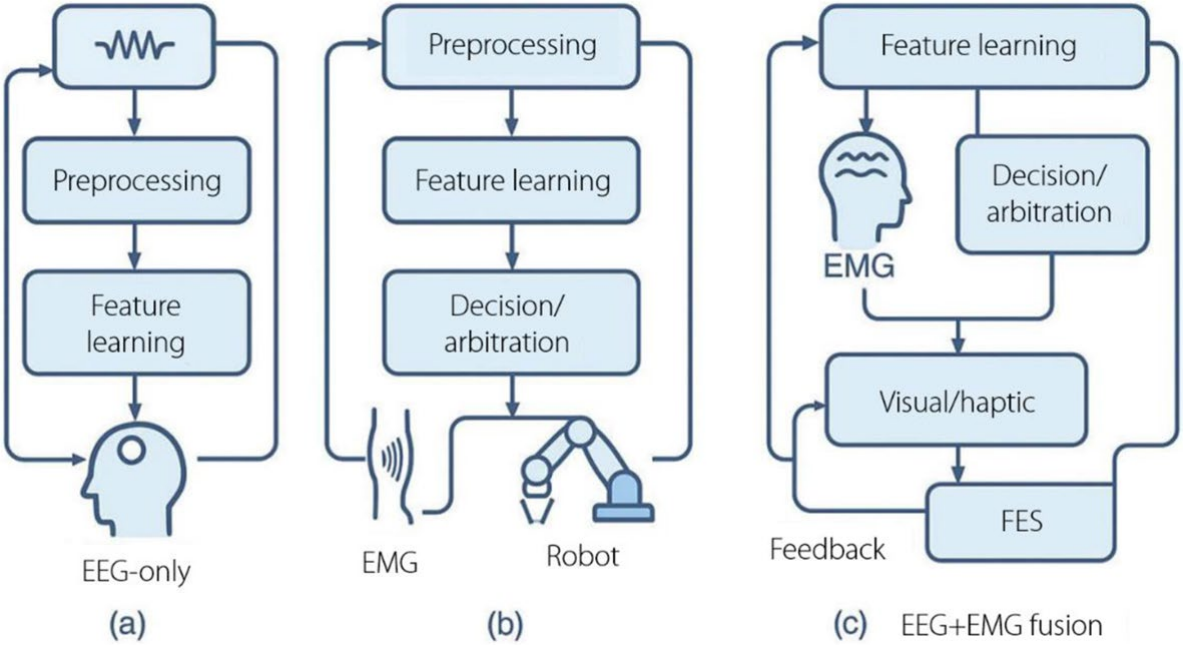
Control principle and system. EEG: Electroencephalography; EMG: Electromyography; FES: Functional electrical stimulation

In robotics, one treats EEG/EMG interfaces as closed-loop controllers rather than as standalone classifiers: neural-muscular intent is encoded by the user, decoded online under explicit latency and safety contracts, mapped to robot control variables (mode, velocity/torque/impedance), and returned via visual/haptic/FES feedback to reduce workload and drift. The system diagram (Fig. 4) instantiates three archetypes–EEG-only, EMG-only, and EEG + EMG fusion–that share a common backbone: synchronized acquisition, preprocessing, feature learning, decision/arbitration, shared autonomy/low-level control, and feedback. Within this loop, EEG is prioritized for sparse, high-level gating (start/stop, mode switch, and target/goal confirmation), given its lower SNR and longer evoked latencies, whereas EMG supports low-latency, continuous modulation of vigor, aperture, and impedance. This separation aligns with the robot control budgets (typical update 50–200 Hz for EMG modulation; 2–5 Hz discrete EEG gating) and maintains explicit stability guarantees (passivity/limits) within the arbitration layer.

EEG encoding and decoders. This work treats the paradigms as encoders that define the discrete symbol set *N* and trial time *T*. (1) MI encodes 2–4 motor-imagery classes (e.g., left/right hand, rest), typically requiring user training and short per-session calibration; decoders emphasize spatial filtering and subject personalization. (2) SSVEP encodes selections by flicker frequency/phase, enabling a larger *N* with minimal training; decoders leverage canonical correlation analysis/filter canonical correlation analysis (CCA/F-CCA) and filter-bank designs that operate at short windows. (3) P300/RSVP encodes row/column or rapid serial selections as ERP detections and trades *N* against the averaging depth to reach reliable P300 peaks.

For reporting, this work recommends the standard information transfer rate (ITR) in bits/minute:

1 $$\mathrm{ITR}=\frac{60}T\lbrack\log_2N+P\log_2P+(1-P)\log_2\frac{1-P}{N-1}\rbrack$$

where (*N, T, P*) is reported for each paradigm and task. In practice, MI often operates with a smaller *N*(2–4) and moderate ITR under the benefit of continuous hold behaviors; P300/RSVP reaches a moderate ITR with minimal user training; SSVEP achieves the highest ITR at short *T* and larger *N*, which is useful for rapid target/goal confirmation. Decoders span common spatial pattern/filter bank common spatial pattern, Riemannian geometry pipelines, linear discriminant analysis (LDA), support vector machines (SVMs), and modern convolutional neural networks (CNNs)/temporal convolutional networks (TCNs)/Transformer backbones; for SSVEP, this paper recommends CCA/F-CCA/filter bank canonical correlation analysis baselines as a must-report comparator.

EMG encoding and decoders. EMG supports both discrete and continuous encoding. Discrete encoding maps a gesture set (e.g., wrist flex/extend, co-contraction) to robot modes, whereas continuous encoding maps amplitude/synergies to speed/torque/impedance. Short per-user calibration (e.g., maximum voluntary contraction normalization, a few repetitions per gesture, 60–90 s total) was stabilized across subjects and dons/doffs. Windows of 100–200 ms with 10–50 ms stride provided responsive updates. Canonical features include time-domain TD-4/TD-6 (mean absolute value (MAV), root mean square (RMS), waveform length (WL), zero crossing (ZC), slope sign change (SSC)), spectral (mean frequency/median frequency (MNF/MDF)), TKE, and wavelet features, and decoders range from LDA/SVM to CNN/TCN/Transformer for end-to-end learning. For synergy-based control, nonnegative matrix factorization or autoencoder bottlenecks produce a low-dimensional command space (6–10 degree of freedom) that maps naturally to humanoid upper-limb poses while respecting the impedance/safety limits. This work advises reporting the command update rate (Hz) and end-to-end latency (ms), along with the accuracy/NRMSE for continuous control.

Training and transmission. Training requirements are paradigm-specific: MI bene-fits from user practice and few-shot recalibration, SSVEP/P300/RSVP requires little user training but necessitates stimulus design and artifact control, EMG needs brief per-session normalization. Transmission capacity should be tied to the robot task using EEG’s higher ITR (with SSVEP/P300) to accelerate goal/target selection or mode switching and EMG’s high-rate continuous channel to maintain smooth closed-loop regulation. For each experiment, reporting (1) *N* (classes/targets), (2) *T* (trial/selection time), (3) *P* (online accuracy), and ITR for the discrete channel, and (4) control-loop rate/latency and task-level outcomes (success, time, safety events, workload) for the continuous channel is recommended. Finally, fusion should be controller-aware: EEG gates autonomy and confirms goals, EMG scales motion vigor/impedance, and arbitration blends both under explicit passivity and rate limits so that intent, autonomy, and safety remain compatible with embodiment.

### Current EEG techniques used in robotics research

**Fig. 5:**
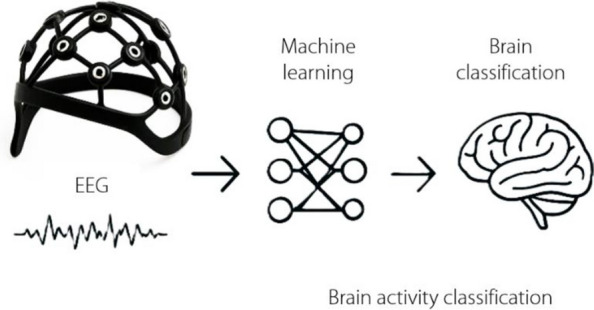
Electroencephalography machine learning

EEG is a powerful neuroimaging tool that measures brain electrical activity. As Fig. 5 depicts, the basic process involves classifying brain activity using EEG signals through machine learning. EEG signals are first acquired to capture temporal neural patterns and then processed using machine learning models (e.g., CNNs, RNNs, or Transformer-based architectures) for feature extraction and pattern recognition. Finally, the classified outputs represent different cognitive states or task intentions. This framework has broad applications in BCIs, cognitive monitoring, and neurorehabilitation. In recent years, EEG has gained significant attention in the field of robotics research, particularly in the development of BCIs for robot control, neurofeedback for HRI, emotion recognition in social robotics, cognitive workload assessment in human-robot collaboration, and neuroergonomics for real-time robotics applications. This review discusses the latest advancements in each of these areas, highlighting the potential and challenges of EEG techniques in enhancing HRIs and control.

#### Machine learning techniques in EEG analysis

Machine learning plays a pivotal role in enhancing the reliability and interpretability of EEG-based robot control. Traditional classifiers such as LDA, SVM, and k-nearest neighbors (KNN) are widely used owing to their simplicity and transparency in low-dimensional MI or SSVEP tasks. However, recent studies have shifted toward deep learning frameworks that integrate spatial and temporal feature learning into a unified model. CNNs exploit spatial covariance across electrode channels to capture topographical patterns of motor-related potentials, whereas recurrent architectures such as long short-term memory (LSTM) and TCN model sequential dependencies in non-stationary EEG signals.

#### BCIs for robot control

BCIs have revolutionized robotics by enabling direct communication between the human brain and robotic systems. Several canonical EEG-based BCI paradigms have been explored for robot control, including MI, P300, steady-state visual evoked potentials (SSVEP), and RSVPs. MI-based BCIs decode motor-related EEG patterns during imagined movements to generate continuous or discrete robot commands [42]. P300-based BCIs exploit stimulus-locked event-related potentials (ERPs) to enable users to select actions or objects displayed on a robot interface or control panel [43]. SSVEP- and RSVP-based BCIs further extend the design space for high-speed and high-accuracy robot control, for example, in multi-degree of freedom robotic arm systems with visual target selection [44, 45]. Recent research has also emphasized hybrid BCIs that fuse EEG with other modalities, such as fNIRS to enhance robustness, improve MI decoding, and support more reliable robot control in complex environments [46, 47]. Along with advanced signal processing and multimodal acquisition pipelines, these paradigms have significantly improved the accuracy, speed, and flexibility of BCI-based robotic control [46].

#### EEG-based neurofeedback for HRI

Neurofeedback techniques utilize real-time EEG feedback to regulate cognitive states during HRIs. Studies have shown that neurofeedback can improve human-robot collaboration by optimizing cognitive workload, stabilizing attention, and enhancing the operator’s sense of agency and control [48]. In shared-control scenarios, neurofeedback has been integrated into robotic exoskeletons and robotic arm systems, allowing continuous alignment of the user’s motor intentions with robotic assistance, thereby improving rehabilitation and assistance outcomes [44, 49]. In parallel, high-speed SSVEP-based and hybrid BCIs–often combined with computer vision, augmented/virtual reality (AR/VR), or eye tracking–provide rich online performance feedback that can be exploited to design adaptive neurofeedback protocols to maintain stable control in demanding tasks [50–52]. Foundational work on SSVEP benchmark datasets, task-related component analysis, dry-electrode implementation, and stimulation waveform optimization has offered detailed guidance for stimulus design and decoding algorithms for neurofeedback-oriented HRI systems [45, 53–55]. Moreover, RSVP and miniature ERP paradigms extend neurofeedback applications to scenarios requiring rapid target detection and adaptive thresholding, enabling the fine-grained adjustment of task difficulty and control policies during interactions [56, 57].

#### EEG in neurological rehabilitation

EEG also plays a significant role in the rehabilitation of patients with neurological diseases by enabling real-time monitoring of cortical activity and facilitating neuroplasticity through feedback-driven training [50, 51]. In post-stroke or spinal cord injury patients, EEG-based BCIs translate MI or event-related potentials into external control signals, allowing patients to reengage impaired neural pathways. Such systems promote motor relearning by coupling intention-related cortical activation with the corresponding robotic or FES feedback. Moreover, adaptive algorithms continuously adjust the stimulus intensity and decoding thresholds according to the user’s cognitive state and fatigue, resulting in personalized and progressively challenging rehabilitation sessions. The integration of EEG with robotic therapy has resulted in measurable improvements in motor recovery, cortical reorganization, and patient engagement, highlighting EEG’s central role in intelligent closed-loop neurorehabilitation.

#### EEG-based emotion recognition in social robotics

Emotion recognition is crucial for developing socially intelligent robots that can interact with humans more effectively. EEG-based emotion recognition techniques have been investigated for recognizing emotional states, such as happiness, sadness, and fear, based on neural activity [58]. Advanced machine learning algorithms, such as SVMs and deep learning networks, have been applied to classify emotional states using EEG signals [59]. These algorithms were applied to humanoid robots to enhance social interactions and create emotionally intelligent robots [60]. EEG-based emotion recognition developments have implications for designing empathetic and emotionally responsive social robots that can enhance HRIs.

#### Cognitive workload assessment in human-robot collaboration

Understanding the cognitive workload of human operators during human-robot collaboration is crucial for optimizing the allocation of tasks and ensuring efficient and safe interactions. EEG has been used to assess cognitive workload levels in various robotic scenarios, such as driving, industrial manufacturing, and collaborative workspaces [61]. Cognitive workload assessments have been applied in diverse scenarios, including robot-assisted therapy and driving assistance [62]. Recent research has focused on developing real-time EEG-based cognitive workload assessment techniques using machine learning algorithms to adapt robot behaviors based on the operator’s cognitive state [63].

#### Neuroergonomics and real-time BCI for robotics

Neuroergonomics focuses on optimizing HRIs by understanding the human brain’s capabilities and limitations. EEG-based neuroergonomic studies have investigated brain responses to different robotic tasks and interfaces, leading to the design of more user-friendly and efficient robotic systems [64]. Neuroergonomics aims to enhance human performance and interactions with complex systems, including robots, by understanding neural mechanisms. Real-time BCI systems integrated with neuroergonomics can adapt robot behavior to human cognitive and emotional states, thereby improving the overall efficiency and safety of HRIs [65]. Recent research has focused on the development of wearable and portable EEG systems for real-time monitoring and interaction with robotic devices.

EEG techniques have shown tremendous potential in robotics research, revolutionizing HRI and control. By understanding and interpreting brainwave patterns, EEG-based BCIs enable direct control of robots through the power of the mind. Moreover, EEG neurofeedback enhances user experience and improves human-robot collaboration. Emotion recognition using EEG in social robotics brings emotionally intelligent robots closer to reality, whereas cognitive workload assessment ensures optimal HRIs. The field of neuroergonomics continues to enhance the efficiency, adaptability, and user-friendliness of robotic systems. As EEG technology advances, its integration into robotics will continue to shape the future of robotics research and applications.

### Current EMG techniques used in robotics research

#### Machine learning techniques in EMG analysis

**Fig. 6:**
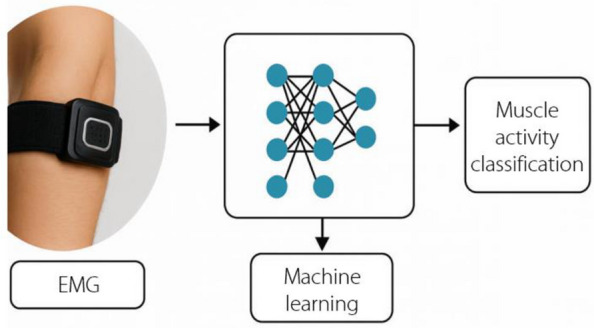
Electromyography machine learning. EMG: Electromyography

The integration of machine learning with surface EMG has been a significant breakthrough in robotics research. As shown in Fig. 6, surface EMG, a noninvasive method, has been paired with machine learning algorithms such as SVM, KNN, and artificial neural networks to achieve accurate signal detection [66]. Furthermore, estimating the muscle force from lower-limb EMG signals is crucial for controlling rehabilitation robots. Recent research has integrated machine learning techniques such as SVM and RF with genetic algorithms for parameter optimization and feature extraction [67]. The resulting output enables the recognition of different muscle movements or gestures, which are essential for applications such as prosthetic control, human-computer interaction, and motor rehabilitation. In addition, a comprehensive review of SVM-based EMG signal classification techniques is presented, summarizing the techniques and accuracies obtained in various studies [68].

#### EMG in human-machine interaction and rehabilitation

Hand gesture recognition based on EMG signals has been a focal point in human-machine interface development [69]. Techniques such as EMG-FMG sensing have shown promise in this domain, facilitating online learning from user’s experience [70]. Moreover, the exploration of the relationship between sEMG and fNIRS has shown positive correlations between EMG and fNIRS signals, especially during dynamic movements [71]. EMG has also been used to detect gait disorders, with various signal processing techniques ranging from traditional statistical tests to complex machine learning algorithms applied for diagnosis.

#### EMG signal processing and EEG integration

EMG signals can introduce distortions in EEG data, making effective investigation and interpretation challenging. Recent research has focused on methods such as Singular Spectrum Analysis and Multimodal Empirical Mode Decomposition to reduce EEG distortion [72]. By combining independent component analysis (ICA) with the wavelet method, researchers have been able to eliminate artifacts without compromising EEG data. In addition, the integration of EMG techniques for the assessment and rehabilitation of motor impairments following stroke has been highlighted, emphasizing the potential of EMG for understanding and treating stroke-induced brain damage [73].

#### Energy expenditure and rehabilitation robotics

Energy expenditure estimation is becoming increasingly important in robotics-based rehabilitation. CNNs have been employed to estimate energy expenditure in both assisted and non-assisted gaits using kinematic and physiological data [74]. In addition, Monte Carlo simulation, a statistical sampling technique, has been applied to estimate elbow angles from EMG signals, especially in post-stroke patients requiring rehabilitation tools based on muscle signals [75]. Furthermore, the potential of robotic interfaces leveraging human-like social interaction techniques has been explored, emphasizing the role of autonomous social robots as companions and professional team members [76].

#### Advanced robotics techniques and tracking

The field of soft robotics has seen exponential growth, especially in wet environments like the sea or in vivo conditions. Inspired by biomimetic sources, researchers have designed soft robots that can perform underwater tasks [77]. In addition, multi-object tracking techniques have been increasingly applied in the navigation tasks of assistive mobile robots to increase the mobility and autonomy of individuals with mobility decline or severe motor impairments [78].

#### Robotics research methods and augmented reality

While not directly related to EMG, the integration of augmented reality (AR) with robotics has been gaining traction. AR provides a new dimension for enhancing HRIs and robotic interfaces [79]. Moreover, methodological paradigms in construction robotics have seen methodological plurality, with researchers increasingly looking at mixed paradigms in data sources and designs, highlighting the evolving nature of research methods in the broader field of robotics [80].

In summary, EEG and EMG are essential neurophysiological tools for robotics. EEG provides insights into cognitive states and mental intentions, enabling brain-controlled robotic applications through BCIs. EMG offers valuable information about muscle activity and control, facilitating intuitive and natural HRIs in prosthetics and exoskeletons. These principles provide exciting possibilities for the development of sophisticated and user-friendly robotic systems that can be seamlessly integrated into various human-centric applications.

### Data analysis of EEG and EMG in robotics

EEG and EMG data analyses play a pivotal role in integrating brain and muscle signals into robotic applications. This subsection presents a comprehensive review of the current EEG and EMG data analysis techniques employed in robotics research. These techniques facilitate the extraction of meaningful information from EEG and EMG signals to enhance HRI, control, and navigation.

EEG and EMG data analysis techniques in robotics research have evolved significantly owing to advances in signal processing, machine learning, and neuroscience. A holistic approach encompassing preprocessing, feature extraction, machine learning, functional connectivity analysis, real-time processing, and ethical considerations will pave the way for successful EEG and EMG integration in robotics. This combined approach enhances the capabilities of HRI, control, and navigation by leveraging brain and muscle signal data.

#### EEG and EMG data acquisition

Data acquisition, which is a critical first step in the application of EEG and EMG signals in robotics, involves capturing the electrical activities of the brain and muscles. This process is performed using specialized hardware such as EEG and EMG electrodes and amplifiers, which detect, amplify, and digitize biological signals for further analysis [25].

**Fig. 7:**
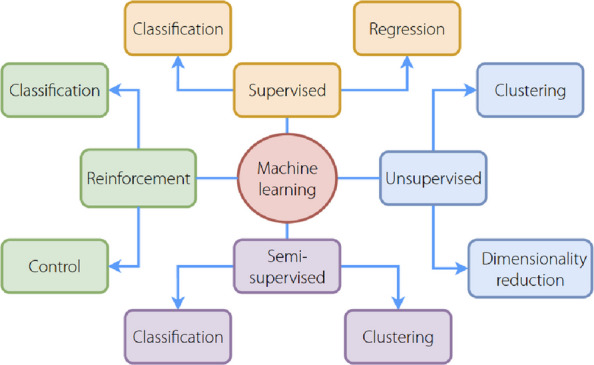
Electroencephalography acquisition loop

For EEG data acquisition (Fig. 7) involves placing a set of electrodes on the scalp according to standardized electrode positioning systems such as the International 10-20 system [81]. Each electrode captures the electrical activity from a specific region of the brain, thereby providing a spatial map of brain activity. This spatial information can be used in robotics for tasks such as controlling the movements of a robotic arm or navigating a robot in a specific direction [82]. During the acquisition of EEG signals, the human scalp cuticle exhibits a high resistance and limited permeability to ions. It is essential to maintain high-quality electrode-scalp contact to reduce impedance and improve the quality of the recorded signals [83].

EEG sensors are used to detect electrical activity in the brain, usually using electrode sensors, which can be classified into noninvasive and invasive electrodes depending on the presence or absence of trauma. Noninvasive EEG electrodes are more popular than their invasive counterparts because of their safety, ease of use, and advances in wearable technology that have been made. Invasive electrodes are less prevalent and primarily employed for the acquisition of high-precision signals in complex cases.

Noninvasive electrodes collect EEG signals from the scalp surface. The electrodes used for scalp EEG can be classified as either wet or dry, depending on whether electrolytes are required on the skin contact surface. Dry-electrode sensors do not require the use of a conductive gel and are therefore suitable for prolonged wear. Wet electrode sensors require the use of a conductive gel, have better signal quality, and are suitable for short-term applications. A review of the literature over the past decade reveals a progressive shift in the development of noninvasive EEG electrodes from wet to dry to semi-dry electrodes.

##### Wet electrode

Wet electrodes use ionic conductors in the form of electrolytic solutions, gels, or pastes to reduce the contact impedance between the skin and the electrode, improving the quality of the recorded signal. They can be broadly classified into four categories: paste, gel, sponge, and semi-dry. In paste-based electrodes, the viscous paste acts as a conductive medium between the skin and electrode. Silver/silver chloride (Ag/AgCl) electrodes combined with paste are the gold standard for EEG recording, excellent for establishing non-polarized electrode/electrolyte interfaces, and preferred in hospitals and laboratories, often with a high signal-to-noise ratio and low electrode-skin interface impedance [84]. Metals such as gold (Au), tin (Sn) and platinum (Pt) are also used in clinical settings, although their DC stabilities tend to be lower than those of Ag/AgCl [85]. In gel-based electrodes, the conductive medium between the electrode and skin is an ionic hydrogel or an organic gel. Gel electrodes are more flexible, dry faster, and are easier to clean than paste electrodes. Modern research on gel electrodes has mainly focused on improving gel materials to reduce skin-electrode contact impedance [86, 87]. Sponges are conductive and are usually soaked in saline solution and placed in contact with the skin to improve ionic conductivity. The evaporation of saline causes a significant increase in the contact resistance of the sponge to the human skin; therefore, sponge electrodes are generally not suitable for long-term measurements. Krishnan et al. [88] developed electrodes made of carbon nanofibers dispersed in hydrophilic polyurethane foam, which maintain a low electrode-skin impedance over time; semi-dry electrodes generally consist of an electrolyte and a porous material that gradually releases the electrolyte onto the skin [89]. They are more convenient to perform and do not require skin preparation. Hua et al. [90] developed a flexible multilayer semi-dry device. Wang et al. [91] proposed a portable semi-dry electrode for EEG data acquisition in long-term driving experiments.

##### Dry electrode

Unlike wet electrodes, dry electrodes do not require gels or plasters. Dry electrodes are easier to use than wet electrodes and can be placed directly on the skin without special preparation. However, a dry electrode cannot be firmly fixed to the scalp, and the signal is more likely to produce motion artifacts. Dry EEG electrodes can be divided into two categories: contact (resistive) and non-contact (capacitive) electrodes. The contact electrode has a variety of shapes, the simplest being the disc electrode, and the best material for making disc electrodes is Ag/AgCl. However, the disc electrode was heavily affected by hair, so other shapes were created; comb, finger, or spike electrodes were designed to easily reach the scalp. Tong et al. [92] investigated the feasibility of 3D printed EEG dry electrodes using conductive wires. The results showed that the alpha wave activity of the subjects could be identified in the frequency range of 20 Hz to 10 kHz. Bristle electrodes have small, brush-like bristles that distribute pressure evenly across the scalp. Microneedle electrodes can penetrate the stratum corneum and greatly reduce the contact impedance. Non-contact electrodes consist of a conductive substrate capacitively coupled to a dielectric or a plate covered with an insulating material [93]. EEG is measured by capacitance; therefore, a dry capacitive or non-contact electrode is not in direct electrical contact with the skin. Hair, air, and insulation inevitably create a high impedance between the conductive layer and the scalp. Therefore, non-contact electrodes are more likely to produce movement artifacts than contact or wet electrodes. A noncontact electrode requires a larger surface area, and the gap between the skin and the electrode must be minimized to reduce the contact impedance [83].

**Fig. 8:**
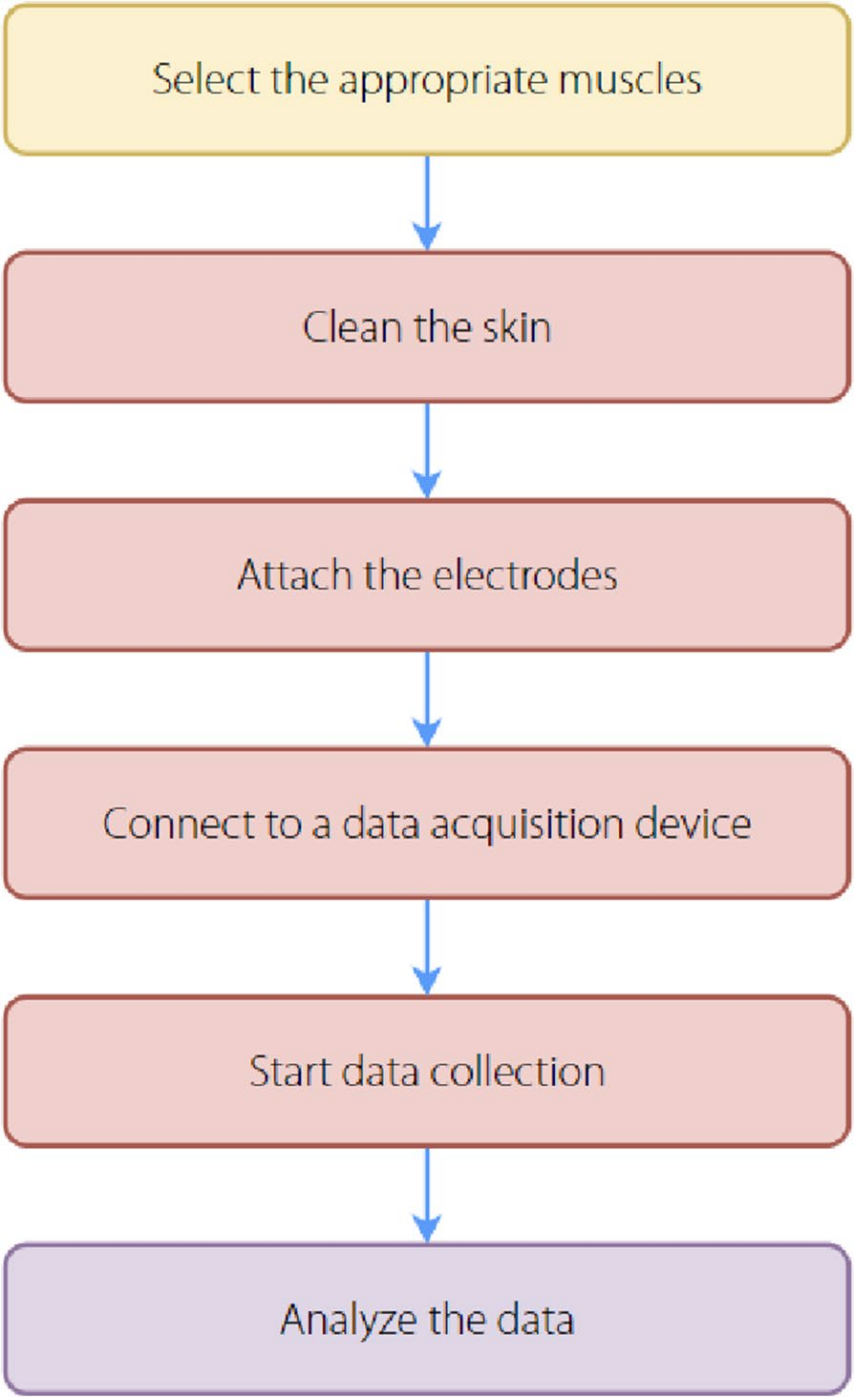
Electromyography data acquisition

For EMG, the data acquisition process (Fig. 8) focuses on detecting the electrical activity produced by skeletal muscles [94]. EMG signal acquisition methods are categorized into invasive and noninvasive types. One is an invasive method, also known as needle electromyography (nEMG), which records electrical activity using piercing electrodes. Piercing electrodes include fine needle and concentric needle electrodes, which are used to measure electrical activity deep within the muscle more accurately. sEMG is another noninvasive method that records muscle activity on the skin surface using surface electrodes. Although nEMG provides more features of muscle activity, it is invasive and cannot be moved or repositioned at will, making it difficult to analyze multiple locations of the target muscle simultaneously. Therefore, sEMG is the preferred method for obtaining the activation time or intensity of superficial muscle signals [95] because it is comfortable and has an extremely low risk of infection in some patients with special conditions [96].

Surface electrodes include the traditional wet electrodes (Ag/AgCl) and various types of dry electrodes. Electrodes are typically placed on skin-overlying muscles. The current gold standard for skin surface biopotential recording involves wet Ag/AgCl electrodes. Similar to the wet electrode used to measure EMG, the Ag/AgCl electrode has an electrolyte gel between the electrode and the skin surface to facilitate ion exchange, increase conductivity, and provide a buffer layer. For dry electrodes, the frontier research direction is focused more on polymer, embroidery, and microneedle array electrodes. Steenbergen et al. [97] proposed conductive elastic electrodes made of polymer materials, such as PEDOT and polyurethane, which combine electrical conductivity, flexibility, and stretchability without the use of conductive adhesives. Sanguantrakul et al. [98] proposed a stretchable dry electrode made of stretchable polydimethylsiloxane and a carbon nanotube composite suitable for long wear and a variety of application scenarios. Etana et al. [99] proposed a textile electrode made of silver-coated polyamide embroidery cotton, copper-nickel-coated polyester, and stainless-steel fibers suitable for embedding in clothing for biological signal monitoring. Xu et al. [100] developed a microneedle array electrode using micromolding technology, which has high reliability and reusability and can effectively reduce the interface impedance and improve the quality of the collected signal.

Electrical signals generated during muscle contraction were detected using these electrodes, amplified, and digitized for subsequent analysis. EMG signals can provide useful information regarding muscle activation patterns that can be used in robotics for tasks such as controlling the grasp of a robotic hand or modulating the gait of a walking robot [101–103].

However, the quality of the acquired EEG and EMG data can be influenced by several factors, including the choice and placement of electrodes, electrical properties of the skin and underlying tissues, and the presence of noise and artifacts from external sources such as power lines or other electronic devices. For different types of EEG and EMG electrodes, the presence of conductive gels, pastes, or electrolytic solutions reduces the contact impedance and improves the accuracy of the data. However, the process of preparing wet electrodes is cumbersome, time-consuming, and requires professional personnel, and conductive gels or adhesives can stain hair and even cause allergic reactions. There is also the possibility of inadvertent diffusion between the gels. High-density recordings can cause short-circuits between adjacent electrodes. The wet electrode must be regularly maintained and cleaned to prevent contamination and degradation by the gel. These disadvantages limit the widespread use of conductive gel-based EEG. Dry electrodes have the potential to increase the popularity of EEG technology and expand their application to long-term monitoring and home EEG assessments. However, the dry electrode lacks an ionic conductive medium, which limits its ability to establish a low-impedance connection with the scalp and is susceptible to motion artifacts, resulting in reduced signal quality compared to the wet electrode. Therefore, careful attention to these factors is needed during data acquisition, and the choice of dry or wet electrodes should be determined based on specific application requirements, signal quality requirements, and the environment to ensure that the recorded signals accurately represent the underlying neural and muscular activity.

#### Transmission of EEG and EMG data

Once the EEG and EMG signals are collected, the data must be transmitted to various end devices such as PCs, cloud servers, or IoT platforms for further processing, analysis, and display. Common communication methods for transmitting EEG and EMG signals include Bluetooth, Bluetooth low energy (BLE), WiFi, and wired transmission, i.e., SPI, I2C and UART, between microprocessors. van den Broucke et al. [104] described a low-power wearable EEG system for auditory brainstem response measurements using SPI data transfer between an ADS1299 chip and an ESP32 microcontroller for data acquisition and storage.

Bluetooth is suitable for devices that require a continuous connection but do not require long battery life, are suitable for short-range wireless transmission, and are widely used in wearable devices and short-range data transmission. Currently, some microcontrollers use Bluetooth for data transmission; for example, Hu et al. [105] used the nRF52832 Bluetooth microcontroller for multichannel EEG acquisition. Kinugasa and Kubo [106] used the EYSKJNZWB (Taiyo Yuden) Bluetooth module as an EMG acquisition system. Bawa and Banitsas [107] developed an EMG system for EMG measurements using commercial dedicated MyoWare, Arduino, and HC-05 Bluetooth modules.

BLE has very low power consumption, can extend battery life, and is suitable for long-term monitoring and data transmission. Battaglia et al. [108] transmitted EEG and EMG signals using the nRF52832 and BLE transport protocols. Enériz et al. [109] used an ARM Cortex M4F microcontroller on an Arduino Nano 33 BLE Sense to implement a low-power EEGNet BCI.

WiFi has high power consumption and is not suitable for long-term battery-powered devices. However, it has a high transmission rate and long transmission distance, which is suitable for applications that require large data transfers, such as cloud data synchronization. Li et al. [110] designed a high-performance multichannel wireless EEG acquisition system that controls a bioelectricity acquisition chip via FPGA (programmable gate array) and transmits it to the host computer in real time via WiFi for display and recording. Zhang et al. [111] used STM32F429 as the microprocessor unit and ESP32C3-MINI1 as the wireless transmitter to wirelessly transmit processed data to the receiving node via MCU. At present, multimodal signal acquisition systems are gradually becoming compatible with the MRI environment for biological signal acquisition, which can simultaneously collect a variety of physiological signals, including EEG and EMG, and ensure the integrity of the signal in a high magnetic field environment through optimized hardware and algorithm processing [112].

Although EEG and EMG signals have different signal processing requirements and application scenarios, they require efficient, low-power, and highly reliable communication methods to ensure data integrity and real-time performance. When transmitting EEG and EMG signals, the application scenario, power consumption requirements, and data rate of the device should be fully considered, and the most appropriate communication mode should be selected to achieve the best performance and user experience.

#### Preprocessing of EEG and EMG data

Once the data have been captured, they undergo a series of preprocessing steps to improve the signal quality and prepare the data for further analysis [12].

For EEG data, preprocessing often includes steps such as filtering to remove noise and artifacts, rereferencing to adjust the signal baseline, and segmentation to divide the continuous EEG data into smaller, more manageable segments. Filtering is particularly important for EEG data because it helps eliminate electrical noise from sources such as power lines, muscle activity, and eye movements, which can significantly distort EEG signals [113]. Techniques include the use of notch filters to eliminate power line noise [114], high-pass filters to remove slow drifts [115], and low-pass filters to eliminate high-frequency noise [116]. In addition, ICA is widely used to separate EEG sources, thereby enhancing the ability to identify brain-related signals [117].

For EMG data, preprocessing typically involves bandpass filtering to isolate the frequency range of interest and rectification to convert the EMG signal into a single polarity [118]. Normalization is often performed to compare the EMG signals across different trials or individuals [119, 120]. This can be particularly useful in robotics applications, where it is often necessary to compare muscle activation patterns across different tasks or conditions.

In both EEG and EMG preprocessing, artifact removal is a crucial step. Artifacts can arise from various sources, including electrical noise, muscle contractions, eye movements, and other non-neural or non-muscular sources of electrical activity. Various artifact removal techniques exist, ranging from simple filtering and thresholding to more advanced techniques based on ICA or wavelet transformations [121, 122].

The choice of preprocessing steps and techniques can significantly influence the quality and interpretability of EEG and EMG data. Therefore, it is crucial to carefully consider the specific requirements and constraints of the application at hand.

#### Feature extraction from EEG and EMG data

Feature extraction from EEG and EMG data is a critical step in data analysis because it facilitates the extraction of the most informative features from raw signals, aiding the conversion of these signals into actionable information [123].

A variety of features can be extracted from EEG data. These features often include different frequency bands such as alpha, beta, theta, and gamma, which are associated with different brain states and cognitive activities. In addition, signal amplitudes and ERPs, which are brain responses directly related to specific sensory, cognitive, or motor events, are frequently extracted from EEG data [124]. Time-domain features such as mean and variance, frequency-domain features such as spectral power, and time-frequency features such as wavelet coefficients are widely used. These features enable the translation of complex EEG recordings into meaningful information for subsequent analysis [125]. More complex features, such as connectivity metrics [126] that measure synchronization between different brain regions, can also be extracted, providing a more holistic view of brain activity. Importantly, the EEG characteristics induced by different paradigms (e.g., MI, SSVEP, P300/RSVP) differ markedly; therefore, the corresponding feature extraction strategies and classification algorithms must be tailored accordingly–for example, emphasizing sensorimotor rhythms in MI paradigms, narrowband spectral peaks in SSVEP paradigms, or time-locked ERP components in P300/RSVP paradigms–rather than adopting a single uniform processing pipeline.

When dealing with EMG data, feature extraction may involve measures such as the RMS or MAV, which provide information about the overall level of muscle activation. Additional features, such as the MDF and MNF, can offer insights into the muscle fatigue state [127]. Furthermore, the WL and ZCs are often used to capture the complexity and frequency content of the EMG signals [123].

It is worth noting that the features to be extracted from the EEG and EMG data are highly dependent on the specific application scenario and research question. Hence, the selection of the most appropriate features requires expert knowledge and an understanding of the underlying neurophysiological and neuromuscular mechanisms. Advanced techniques such as machine learning can aid in this process by automatically selecting and optimizing the feature set based on task requirements and available data.

Therefore, feature extraction serves as a critical bridge between the raw EEG and EMG data and the subsequent stages of data analysis. The extracted features provide a foundation for the interpretation and application of these signals in robotics.

#### Interpretation of EEG and EMG data

The interpretation of EEG and EMG data is the final step in translating distilled features into meaningful information, thereby establishing a direct connection between neurological or muscular activity and robot control commands.

Various statistical and machine learning algorithms are commonly used to interpret and classify extracted features [128]. These include LDA, SVM, neural networks, and decision trees. Each method offers distinct advantages and trade-offs in terms of complexity, computational load, and performance. The choice of an appropriate method often depends on the specific application, nature of the data, and task requirements.

For instance, SVMs, which aim to find the optimal hyperplane to separate different classes in a high-dimensional feature space, have been widely applied because of their robust performance in high-dimensional nonlinear classification problems. However, neural networks are particularly useful when dealing with large, complex datasets because they are capable of learning intricate patterns and relationships between features. Sequential pattern-mining techniques, such as hidden Markov models, aid in recognizing temporal patterns within EEG signals and enhancing the prediction of user intentions [129].

**Fig. 9:**
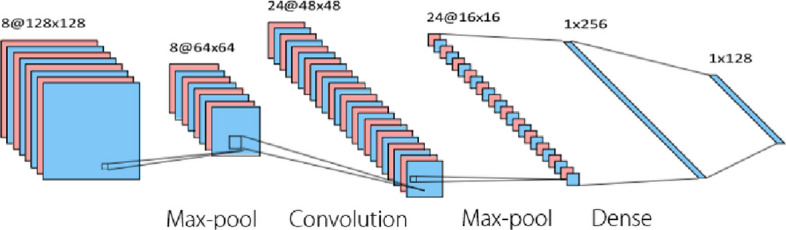
Convolutional neural networks

**Fig. 10:**
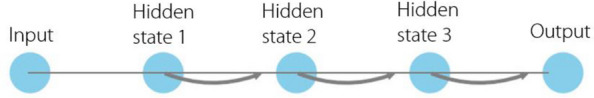
Recurrent neural networks

**Fig. 11:**
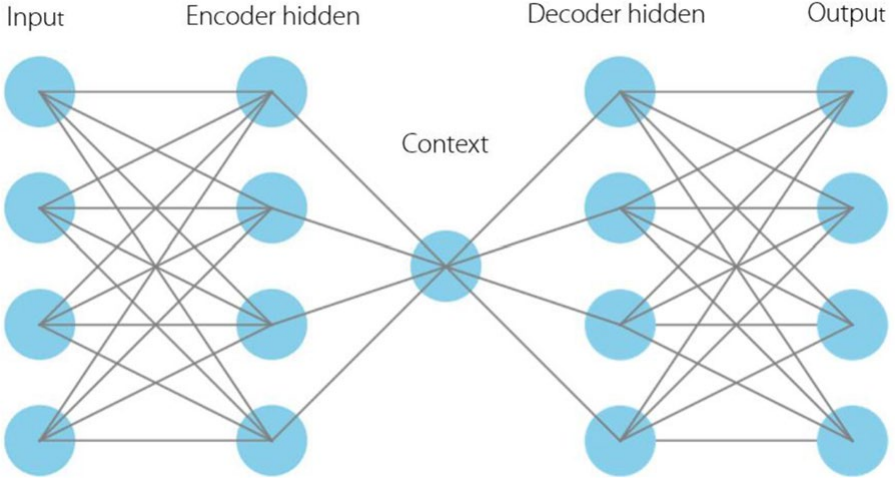
Encoder-decoder architecture

In recent years, as shown in Figs. 9 and 10, deep learning techniques [130] such as CNNs and recurrent neural networks (RNNs), have emerged as powerful tools for EEG and EMG data interpretation. These techniques can automatically learn features from raw or minimally preprocessed data and provide high classification accuracy, making them particularly attractive for real-time applications [128, 131]. CNNs extract spatial features through convolution, pooling, and dense layers for high-level classification, whereas RNNs capture temporal dependencies across hidden states to model dynamic signals, such as EEG, EMG, or speech. Both architectures are widely used in image processing, biosignal analysis, and intelligent control. There have also been studies utilizing an encoder-decoder architecture (Fig. 11) for signal noise reduction. It consists of an input layer, hidden encoder layers, a context vector, and hidden decoder layers. The encoder extracts high-level representations from the input sequence and compresses them into a context vector, whereas the decoder generates a corresponding output sequence based on contextual information. This architecture is widely used in sequence-to-sequence tasks such as machine translation, speech recognition, and EEG/EMG-to-command mapping.

Moreover, the interpretation of EEG and EMG data can be enhanced using hybrid or multimodal systems. By combining information from different sources, these systems provide accurate and reliable control signals for robotic applications [132].

The interpretation of EEG and EMG data plays a pivotal role in transforming neurophysiological and muscular signals into usable inputs for robotic systems. Continued advancements in machine and deep learning techniques are expected to enhance the interpretability and usability of these signals in robotics.

Functional connectivity analysis was used to examine the correlations and interactions between different brain regions. Graph-theory-based approaches characterize brain networks and identify key regions involved in specific tasks. This analysis assists in understanding the neural mechanisms underlying cognitive processes and improves the accuracy of robotic control [133].

Real-time EEG data processing is essential for responsive robotic applications. Online algorithms, such as adaptive filtering and incremental learning, enable the immediate interpretation of EEG signals, which are crucial for the real-time control of robots [134].

With advances in EEG data analysis, ethical considerations have become paramount. Ensuring user privacy, data security, and informed consent are essential when implementing EEG-driven robotics. To address these concerns, researchers must employ techniques for anonymization, secure data storage, and transparent communication [135].

### Human-side and robot-side signal analysis

Robotics are structured mirrors of the human side–measurable joint states, motor currents, force/torque and tactile signals, and semantic perception–which close the loop with human intent. This asymmetry relocates uncertainty: human signals are non-stationary and user-dependent, with labels that often refer to latent intentions (e.g., imagery, attention, effort) and thus require calibration, normalization, and continual personalization across sessions; robot signals are comparatively repeatable and strongly labeled by controllers and estimators, but they inherit embodiment-dependent shifts whenever tools change, contacts vary, or autonomy layers update their policies. Latency plays a different role on both sides: EEG is best treated as a discrete gate for task-level decisions, EMG as a continuous modulator, and the robot control stack enforces passivity, rate limits, and predictive safety so that human-in-the-loop delays do not jeopardize stability. As a result, analysis pipelines must be co-designed. Intention decoding should be parameterized by the robot’s admissible action space and safety envelopes, and robot-side estimators should, in turn, expose the uncertainty and affordances that simplify human intent decoding. When success criteria are expanded from offline accuracy to closed-loop competence–task completion, near-miss rate, recovery after perturbations, and operator workload–the differences between “human-side analysis” and “robot-side analysis” become complementary rather than contradictory, yielding systems that adapt simultaneously to people and embodiments.

## Applications of EEG and EMG in robotics

EEG and EMG signals play crucial roles in enhancing HRI. EEG-based BCIs enable direct communication between the human brain and robots, allowing users to control robotic movements through imagined tasks or MI. EMG-based gesture recognition enables the intuitive control of robotic prosthetics or exoskeletons, offering individuals with mobility impairments enhanced dexterity and mobility. The integration of EEG and EMG signals into robotic systems has paved the way for innovative applications that merge intuitive human control with robotic precision and automation. Table 1 summarizes the main aspects of EEG and EMG applications in robotics.

**Table 1:** Representative applications of electroencephalography and electromyography in robotics

EEG/EMG	Application	Description
EEG	BCI for robot control	Direct control of mobile, assistant, or exploration robots using decoded brain activity [136]
EEG	Cognitive state monitoring	Passive BCIs monitor workload or attention so that collaborative robots can adapt motion speed, shared-autonomy level, or task allocation in real time [137]
EEG	Neurofeedback systems	Robot-assisted rehabilitation systems provide real-time EEG-based feedback to shape motor relearning, for example, neurofeedback training combined with shoulder exoskeletons in post-stroke patients [138]
EEG	Assistive robots	EEG-driven assistive robots, such as P300-based upper-limb assist devices, translate users’ intentions into manipulation and ADL support, linking neural signals to assistant and medical robotics [139]
EMG	Prosthetic limb control	EMG-based pattern-recognition and pro-portional control enable intuitive actuation of robotic prosthetic hands and wrists [140]
EMG	Exoskeletons for rehabilitation	EMG-driven upper-limb exoskeletons and hybrid EMG-FES systems support motor recovery by assisting or resisting movement according to residual muscle activity [141]
EMG	Gesture-based control	High-density EMG and robust gesture-recognition algorithms allow hands-free control of physically assistive mobile manipulators and other assistant robots [142]
EMG	Strength augmentation	EMG-based motor-intention prediction is used in industrial and wearable exoskeletons to scale or share load with the user, supporting biomechanics research and strength augmentation [143]

*EEG* Electroencephalography, *EMG *Electromyography, *BCI* Brain-computerinterface, *FES* Functional electrical stimulation

In HRI, EEG and EMG interfaces have transformed the conventional communication paradigms, offering an intuitive and direct link between humans and robots [34, 144]. Users can subtly instruct robotic systems using their neural or muscular signals, culminating in fluid and natural interactions. Notably, EEG-based HRI exhibits a higher level of cognitive engagement [145], whereas EMG interfaces allow more kinetic interactions [146].

Robot control is another primary domain in EEG and EMG applications. EEG-driven robots convert users’ cognitive states into specific commands, making them effective tools in areas ranging from industrial automation to personalized home assistance. By contrast, EMG-based robots rely on muscle activity, demonstrating exceptional performance in prosthetic control, where the user’s residual muscle signals dictate the robot’s movements [147–149]. Comparative studies suggest that, while EEG provides a broader range of control options, EMG ensures a more instantaneous and forceful response [6].

In the realm of navigation, EEG and EMG interfaces have offered breakthroughs in controlling mobility devices such as robotic wheelchairs and drones. EEG-based navigation systems generally rely on the brain activity associated with different cognitive tasks [150, 151], whereas EMG-based systems may utilize facial muscle movements to steer the device [152]. Here, EEG interfaces offer more diverse control, whereas EMG systems exhibit faster response times.

EEG and EMG offer distinct advantages in robotics, such as direct control, non-invasiveness, and real-time operation, fostering a more immersive and engaging user experience. However, these techniques have certain limitations. Although EEG signals are rich in control possibilities, they suffer from low spatial resolution and are prone to noise and artifacts [153]. Conversely, EMG signals, while providing strong and swift control, can be influenced by muscle fatigue and individual variability, and require a certain level of muscular control from the user [154].

### Principal research and key applications of EEG and EMG in robotics

The key applications discussed in this subsection cover a diverse range of areas, including communication for locked-in patients, neurorehabilitation, HRI, robotic arm control, Parkinson’s disease treatment, emotion recognition, wheelchair control, prosthetics and orthotics, data augmentation, and fusion. Each study contributes valuable insights into the growing field of EEG and EMG robotics applications, demonstrating the potential of this technology in enhancing the functionalities of robotic systems and improving the quality of life of individuals with various physical disabilities and neurological conditions. As EEG and EMG research progresses, these applications will continue to shape the future of robotics and assistive technologies, positively impacting society and healthcare [155, 156]. The main aspects involved are as follows:

#### BCI communication in robot control

One key area of research in the field of EEG in robotics focuses on developing BCIs for direct robot control using MI-based EEG signals. Chaudhary et al. [157] explored the use of EEG-based BCIs to facilitate communication between patients in a completely locked state. This research is significant for individuals with severe motor disabilities, as it provides them with a means to communicate and interact with robotic devices through MI-based EEG signals. López-Larraz et al. [158] investigated the EEG decoding of motor attempts and MI in patients with cord injuries. Researchers have demonstrated the potential of EEG robotics applications to enable direct robotic control through user intentions, thereby presenting a significant step forward in assistive robotics.

Wang et al. [159] proposed a robust MI decoding approach using a combination of sparse logistic regression and decision boundary thresholding to achieve real-time control of a robotic arm through MI tasks. Zhang et al. [160] presented a novel SSVEP-based BCI system for controlling robotic platforms using velocity modulation. The system utilizes flickering stimuli of varying brightness to modulate the velocity of the robotic arm, thereby offering an enhanced level of control in BCI applications.

Hybrid BCIs, which combine EEG with other neuroimaging techniques, have shown promise for advancing assistive robotics. Jayaram et al. [161] reviewed the implications of brain-computer interfacing in amyotrophic lateral sclerosis and its potential for functional and communication rehabilitation. The integration of EEG with other modalities, such as fMRI and ECoG, enables more comprehensive information about brain activity during HRIs, thereby improving the capabilities of brain-controlled robotic systems. Li et al. [162] proposed a hybrid BCI system that combined P300 and SSVEP signals for wheelchair control. This system enhanced the independence and mobility of users with motor impairments, showcasing the potential of EEG robotic control in assistive robotics.

#### EEG-based rehabilitation

Palmarini et al. [163] conducted a systematic review on the integration of EEG and AR in neurorehabilitation. The study focused on how EEG robotics applications, coupled with AR environments, can enhance motor recovery and functional outcomes for patients with neurological disorders. A systematic review [163] examines the integration of EEG and AR in neurorehabilitation. This study highlights the potential of EEG robotic applications in enhancing motor recovery and functional outcomes in individuals with neurological disorders. AR environments paired with EEG-based BCIs have shown promising results in providing interactive and engaging rehabilitation interventions.

Another significant research area involves the use of EEG-based BCIs to control robotic exoskeletons in rehabilitation [164]. By decoding motor intentions from EEG signals during MI tasks, stroke survivors and individuals with motor impairments can regain movement control using robotic exoskeletons. The integration of EEG-based BCIs with exoskeletons offers a potential pathway for personalized and intensive neurorehabilitation.

#### Brain connectivity patterns

Understanding the neural mechanisms underlying HRIs is essential for developing socially engaging robotic systems. Khan et al. [165] found that robotic hand training significantly facilitated stroke motor recovery. This was evidenced by changes in brain connectivity and electrical patterns, indicating neuroplasticity changes. Some researchers [166] have investigated band-specific alterations in cortical connectivity in early Parkinson’s diseasePD and their clinical correlations.

High-density EEG was used to assess resting-state functional connectivity abnormalities in patients with early stage Parkinson’s disease and examine their relationship with motor and non-motor Parkinson’s disease symptoms.

#### EEG-based emotion recognition for HRI

Li et al. [167] explored emotion recognition using EEG brain networks. This presents a novel approach for identifying discriminative graph topologies within EEG data to enhance emotion recognition accuracy. This paper proposes a model that learns these topologies and demonstrates its effectiveness through various experiments. Another recent study explored the development of real-time emotion recognition systems using EEG signals to enable more empathetic and responsive HRIs [168]. By analyzing the EEG patterns associated with different emotional states, the robotic system can adapt its behavior and responses to better engage and understand human users. Such advancements hold great promise in enhancing the social acceptance and effectiveness of robots in various applications, including healthcare and companion robotics.

#### Hierarchical control framework for brain-robot interface

Zhang et al. [169] discussed self-reconfigurable hierarchical frameworks for controlling robot swarms. They introduced a systematic approach to construct and adapt these frameworks, ensuring their rigidity and hierarchy using only local information. This method enhances the management and scalability of large robot swarms, allowing them to dynamically adjust their structures for various tasks or respond to unexpected changes in the environment.

#### Integration of eye-tracking and eeg in robotics and assistive technologies

The integration of eye tracking and EEG has become a powerful strategy for enhancing interaction bandwidth and usability in robotics and assistive technologies. A series of hybrid BCI systems have demonstrated that combining gaze information with EEG, particularly SSVEP responses, can support the flexible and high-speed control of robotic devices and virtual environments. For example, Guo et al. [170] proposed a robotic arm control system with simultaneous and sequential modes that combined eye tracking with SSVEP cues in a VR environment, whereas Tan et al. [171] developed an autonomous hybrid BCI that integrated EEG and eye tracking for interaction in virtual environments. Ma et al. [172] further demonstrated that coupling BCI with eye tracking enables high-speed text entry in VR, highlighting the importance of gaze-contingent stimulus selection and attentional focusing in multimodal interfaces. These developments complement the work of Kim et al. [173], who systematically compared VR teleportation methods that integrate hand-tracking, eye-tracking, and EEG to assess efficiency, accuracy, and usability in VR locomotion and built on foundational EEG-based BCI research on high-speed SSVEP paradigms and benchmark datasets [50, 53]. In parallel, Punsawad et al. [174] introduced an EEG-based BCI that leverages illusory visual-motion stimuli to enhance MI for assistive communication, demonstrating that carefully designed visual and oculomotor engagements can substantially improve the practicality and effectiveness of EEG-based interfaces for users with severe motor impairments.

#### Neurofeedback training in robotic rehabilitation

EEG-based neurofeedback training is a promising direction in robotic rehabilitation settings [175]. Patients can receive real-time feedback regarding brain activity during rehabilitation exercises, thereby facilitating brain plasticity and motor-skill improvement. The combination of robotic devices and neurofeedback enables adaptive and individualized therapy plans for better rehabilitation outcomes (Fig. 4). Guggenberger et al. [176] investigated BMI for stroke rehabilitation by comparing robotic orthosis with FES. It involved 20 healthy participants and assessed the mental, physical, and temporal demands of controlling a BMI through MI. The results indicated that both robotics and FES posed similar workloads, with the mental demand being the most significant.

#### Decoding human intention for robot navigation using EEG

Decoding human intention for robot navigation using EEG signals is another area of active research. Koo et al. [177] explored the use of a hybrid near-infrared spectroscopy –EEG system for self-paced BCIs during online MI tasks for robot navigation. This study demonstrated the feasibility of using EEG-based BCIs for intuitive and efficient robot navigation, offering opportunities for enhancing autonomous and brain-controlled robotic systems.

#### HRI

For EMG, it has emerged as a pivotal tool in the realm of robotics, offering a direct interface between human muscle activity and robotic actions. EMG provides a direct window into human intentions, enabling robots to interact, respond, and navigate with heightened intuition and precision. With the advent of conductive textiles, there is the potential for more user-friendly and wearable EMG systems that enhance both comfort and signal accuracy [178]. In the field of HRI, EMG has unlocked new dimensions of empathy and responsiveness. Robots equipped with EMG sensors can ‘sense’ human muscle movements. For instance, dynamic hand gesture recognition systems, such as those proposed by Kim et al. [179], leverage EMG signals to control industrial robots in real time. Furthermore, Zhou et al. [180] investigated the frequency behavior of passivity maps for the upper limb using visual electromyographic feedback, enhancing the understanding of human intentions during physical interactions with robots . Another significant contribution by Aarthy et al. [181] combined depth vision learning with EMG-based hand gesture detection, offering a unique strategy for HRI.

#### Robot control

The realm of robot control has been revolutionized by EMG interfaces. Chen et al. [182] described an architecture applied to industrial robots for healthcare applications in which EMG and force sensor data were utilized for robot control. In another study by Ma et al. [74], deep learning tools were employed to estimate the energy expenditure in assisted and non-assisted gait using EMG wearable sensors, demonstrating the potential of EMG in robotic-assisted movements. Furthermore, some research focuses on achieving motion-robust epidermal signal monitoring, brain-body interaction mechanisms, and neuroprosthetic strategies that enhance motor control and rehabilitation. These provide a solid foundation for EMG-based robotic control [183–186]. 

#### Navigation

EMG’s direct application in navigation might seem less intuitive than in HRI or control; its indirect contributions are profound. Advanced artificial intelligence (AI)-driven navigation models such as the visual language maps proposed by Huang et al. [187] can be informed by human intentions captured through EMG, making robots more adept at navigating complex environments. Shah et al. [188] introduced a general navigation model that could be trained on diverse robot data, showcasing the potential of integrating EMG data for broader robot navigation applications.

#### Related machine learning methods

1. Feasibility of EMG-driven orthoses/prostheses. Research has shown that surface and intramuscular EMG can reliably actuate orthotic and prosthetic devices, enabling intuitive assistive control by decoding muscle intent [189].
2. Force estimation using deep learning. Integrating deep learning with EMG–e.g., 1D/2D CNNs for spatiotemporal features, TCN, LSTM/GRU for sequence modeling, and transformer encoders with attention–has improved continuous force/torque estimation accuracy and robustness in EMG-driven systems [190–192].
3. Synthetic data for the low-sample regimes. To mitigate the limited data and subject variability, synthetic EMG generation using sequence models (e.g., GPT-2 adapted to biosignal token streams) augments training sets and yields gains in gesture recognition under few-shot and cross-session scenarios [193].
4. Pre-processing and feature engineering. Robust pipelines emphasize band-pass/notch filtering, artifact reduction, and windowing, followed by multigranular features: time domain (RMS, MAV, ZC, SSC, WL, AR coefficients), frequency domain (PSD, MF/MNF/MDF), and time-frequency (STFT, wavelet packet energies, EMD/CEEMDAN components). Feature selection (mRMR, ReliefF, LASSO) reduces redundancy and overfitting [194, 195].
5. Personalization and online calibration. Personalized calibration–within- and across-session normalization, domain adaptation, drift compensation, incremental learning, and few-shot recalibration–improves stability and portability under day-to-day electrode shifts and physiological changes [194, 195].
6. Multimodal fusion (EMG + EEG). The combination of EMG with EEG integrates complementary motor planning and muscle execution. Early feature concatenation, late decision fusion, and cross-modal alignment/attention mechanisms enhance the robustness and accuracy of movement identification and robot control, particularly under low SNR and task switching [196].
7. Non-deep learning baselines (methods without deep networks). A substantial body of EMG work employs classical models such as LDA/QDA, SVM (linear/RBF), kNN, Naïve Bayes, random forests (RFs)/gradient boosting, sparse representation classifiers, hidden Markov models, and template-based DTW. Paired with handcrafted features, these methods train quickly, are interpretable, and remain strong baselines in small samples or resource-constrained settings [194, 195].
8. System integration and deployment. End-to-end systems integrate low-latency acquisition and filtering, robust feature/encoding, personalized calibration, continuous state estimation, and intent-to-command mapping (thresholding/state machines/optimal control), thereby enhancing natural interactions in orthoses, prostheses, and assistive/rehabilitation devices [189–196].

### Recent progress on humanoids with EEG/EMG interfaces

Recent studies on humanoid platforms have converged toward hybrid intent pipelines in which EEG triggers or confirms high-level goals, while EMG–often fused with inertial measurement unit signals–continuously modulates upper-limb trajectories and grasp impedance, enabling robust reaching-grasping with fewer false activations. Low-dimensional muscle-synergy mappings further compress EMG control spaces into a handful of interpretable synergies aligned with humanoid kinematics, reducing the calibration overhead without sacrificing dexterity. At the behavioral level, shared autonomy has matured from simple assistance to intent arbitration that integrates onboard perception and grasp planners, allowing the humanoid to propose safe grasps and trajectories while the operator approves, overrides, or scales motion vigor through biosignals. Evaluation practices have also shifted from dataset accuracy to task-facing metrics–handover success, completion time in clutter, contact safety envelopes, and NASA-TLX scores–making the results more comparable and operationally meaningful. Finally, with consistent haptic or FES feedback, operators exhibit improved embodiment and reduced co-contraction, and lightweight few-shot recalibration maintains stable performance across sessions and don/doff events. Together, these trends suggest that the value of EEG/EMG for humanoids lies less in decoding ever more labels and more in structuring the decision authority between humans and autonomy to achieve robust manipulation in contact-rich scenes.

## Challenges and future directions in the current era of artificial intelligence

The intersection of EEG and EMG with robotics has ushered in significant advancements in this field. Yet, it’s important to recognize that this fusion also comes with unique challenges that require intensive research for further optimization. Similarly, the future prospects of this synergistic alliance hold tremendous potential, warranting closer inspection.

Several challenges exist for the seamless integration of EEG and EMG with robotics. For instance, EEG signals are prone to artifacts and noise interference because of their low signal-to-noise ratios. This situation is further complicated by their low spatial resolution, which makes it difficult to pinpoint the origin of the neural activity accurately.

In contrast, EMG signals, while offering better spatial resolution, can be affected by physiological factors, such as muscle fatigue and crosstalk from neighboring muscles. Furthermore, the inherent variability of EMG signals among individuals adds another layer of complexity, necessitating personalized calibration procedures for each user.

In addition to these technical challenges, ethical and safety considerations must be addressed. Because EEG and EMG interfaces probe users’ physiological signals, it is imperative to ensure privacy and data security. Similarly, safety measures must be in place to protect users from potential harm due to malfunctioning robots or inappropriate interpretation of EEG and EMG signals. The main challenges in the current era of AI and deep learning are as follows:

### Strong signal randomness

EEG signals are inherently noisy and susceptible to interference from various sources, including muscle activity, eye movement, and environmental electrical noise. Similarly, EMG signals, which measure muscle electrical activity, are prone to noise from sources such as adjacent muscles, movement artifacts, and external electrical interference. For both EEG and EMG, robust preprocessing techniques such as adaptive filtering and artifact removal are essential for mitigating signal noise and enhancing the quality of data for AI applications.

### Inter-individual variability

Inter-individual variability refers to the differences in EEG patterns among different individuals. These variations can arise from anatomical differences, scalp thickness, or neural network variations. Similarly, EMG patterns can vary among individuals owing to muscle fiber distribution, muscle thickness, and neuromuscular junction properties. In the context of robotics and AI, where standardized models are desired, this variability poses a challenge. Subject-specific calibration and adaptive algorithms are necessary to account for these differences in EEG- and EMG-based systems.

### Real-time processing demands

Real-time processing of EEG and EMG data is crucial for responsive human-robot interaction and control. The complex nature of these data analyses, coupled with the need for low-latency responses, presents computational challenges. Balancing the need for accurate signal interpretation with real-time processing requirements requires efficient algorithms, optimized deep learning models, and dedicated hardware resources.

### Data volume and dimensionality

Both EEG and EMG data can be voluminous, comprising multiple channels sampled at high frequencies. These high-dimensional data place a strain on computational resources, especially in deep learning frameworks, leading to increased processing times. Dimensionality reduction techniques such as principal component analysis, feature selection, and autoencoders are necessary to manage data complexity and facilitate efficient analysis in the AI era.

### Cognitive and muscular state ambiguity

Decoding cognitive states solely from EEG signals can be challenging due to the ambiguity of brain patterns. Similarly, interpreting muscle states from EMG data can be complex because different muscle actions may lead to similar EMG patterns. Advanced machine learning algorithms that capture temporal patterns and context and integrate multimodal data (combining EEG and EMG) are required to improve the accuracy of state recognition in both the cognitive and muscular domains.

### Ethical and privacy concerns

The integration of EEG and EMG in robotics and AI raises ethical questions related to user consent, data ownership, and privacy. The extraction of cognitive, emotional, and muscular information from these signals raises concerns regarding the potential misuse of personal data, especially with deep learning models that can extract intricate patterns. Addressing these ethical concerns, including transparent data handling and user awareness, is crucial to ensure responsible and secure EEG- and EMG-driven robotics and AI.

Despite these challenges, the future of EEG and EMG in robotics holds great promise. Advancements in machine learning algorithms and signal-processing techniques may help overcome some of the current limitations by enabling more accurate extraction and interpretation of EEG and EMG signals.

Furthermore, the advent of hybrid BCI systems that combine EEG and EMG signals can exploit the strengths of both techniques, leading to a more robust and flexible control of robotic systems [171, 197]. For example, EEG signals can be used for high-level decision-making tasks, while EMG could handle more immediate and forceful commands.

**Fig. 12:**
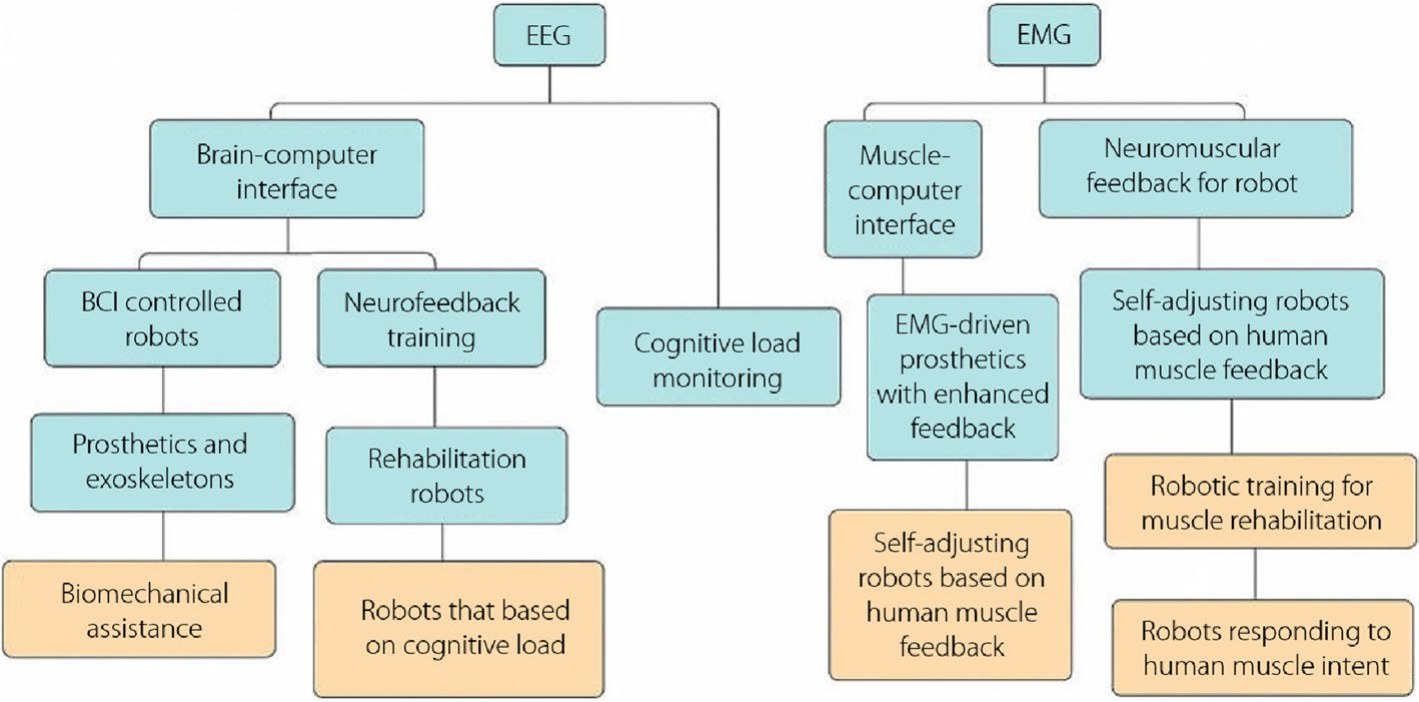
The future direction of electroencephalography and electromyography.  EEG: Electroencephalography; EMG: Electromyography

EEG, which is a cornerstone for understanding cerebral activity, has profound applications in BCIs, neurofeedback training, and cognitive load monitoring (Fig. 12). These domains have the potential to create robots that not only understand but also adapt to human cognitive states. In contrast, EMG, which captures the essence of muscular electrical activities, underpins the evolution of muscle-computer interfaces, neuromuscular feedback systems, and adaptive learning mechanisms (Fig. 12). The intrinsic value of these technologies lies in their capability to forge a responsive and symbiotic relationship between humans and machines, as exemplified by innovations such as EMG-driven prosthetics and biomimetic robots.

Emerging fields such as neuromorphic engineering [198], where artificial systems are designed to mimic biological neural systems, may also offer novel solutions for enhancing the integration of EEG and EMG with robotics. Further research is required in the areas of user training and interface design to make EEG- and EMG-controlled robots more user-friendly and accessible to a wider population, including people with disabilities. Possible future research directions are discussed below.

### Multi-modal sensor fusion

Future research could focus on integrating EEG data with data from other sensors, such as eye-tracking, EMG, and heart rate monitors [199]. For EMG, combining its data with other modalities can enhance our understanding of muscle activity in relation to cognitive state. This multimodal fusion can enhance the accuracy and reliability of both cognitive and muscular state recognition, enabling more robust human-robot interaction and control.

### Adaptive algorithms and transfer learning

Developing adaptive algorithms that can quickly adapt to changes in EEG and EMG patterns or user characteristics is crucial. Future research could explore transfer-learning techniques that leverage data from multiple users to improve the generalization of both EEG- and EMG-based models, thus reducing the need for extensive user-specific calibration and enhancing the adaptability of robotic systems [129].

### Real-world applications and user-centered design

Researchers could focus on deploying EEG and EMG-driven robotic systems and AI applications in real-world scenarios. For EMG, this could indicate more intuitive prosthetic control or rehabilitation exercises. User-centered design principles can guide the development of interfaces that seamlessly integrate EEG and EMG technologies into everyday tasks, thereby enhancing user experience and accessibility [200].

### Cognitive state decoding for enhanced control

While EEG focuses on cognitive states, EMG provides insights into muscular intentions. Future studies could delve into decoding muscle activation patterns in tandem with cognitive processes such as decision-making. This combined approach can lead to more sophisticated robotic control strategies that align with users’ cognitive states, intentions, and physical actions [201].

### Neurofeedback and BCI training

Beyond EEG-based neurofeedback, research could explore EMG-based feedback systems that help users modulate their muscle signals effectively. This could be particularly beneficial in rehabilitation settings where users need to relearn muscle control. Optimal training protocols for combined EEG-EMG BCIs can further accelerate user proficiency and enhance interaction quality [202].

### Cross-domain applications

Extending EEG and EMG-driven robotics to cross-domain applications offers vast potential. For EMG, applications in sports training, physiotherapy, and musical instrument control can be explored. Combining EEG and EMG in VR, gaming, or assistive technology can lead to more immersive and responsive experiences, catering to both cognitive and physical user inputs [203].

### Ethics and privacy frameworks

With the integration of EMG data, the ethical considerations expand. EMG can reveal information about a user’s physical health, muscle condition, and fatigue level. Future research should focus on developing guidelines that ensure responsible data usage, informed consent, and the protection of user privacy, considering both EEG and EMG data in robotics and AI applications [204].

The future of EEG in robotics and AI is ripe with possibilities. Multimodal sensor fusion, adaptive algorithms, real-world applications, enhanced cognitive state decoding, neurofeedback, cross-domain applications, and ethical considerations are only a few areas in which researchers can contribute to shaping the landscape. By addressing these research directions, the field can unlock the full potential of EEG-driven technologies, resulting in more seamless and intuitive human-robot interaction and control.

## Conclusions

The integration of EEG and EMG into robotics is a critical aspect of the trajectory of interdisciplinary research. These neural interfaces bridge the divide between humans and machines and offer an unprecedented level of interaction and control. This paper has endeavored to provide an overview of the principles, techniques, applications, and data analysis methods associated with the use of EEG and EMG in robotics and illuminates the challenges faced and potential future directions.

**Fig. 13:**
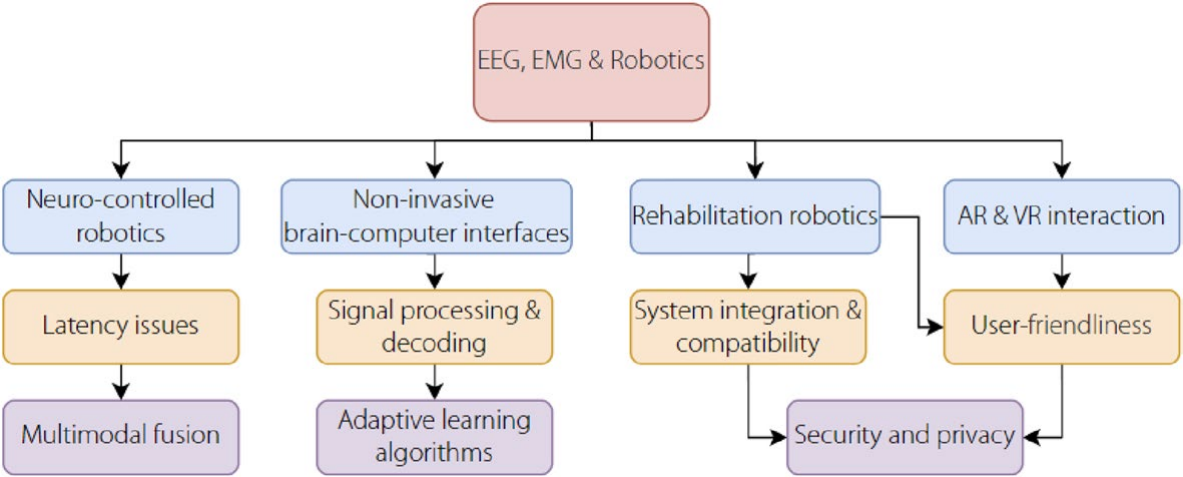
The future direction of electroencephalography, electromyography and robotics. EEG: Electroencephalography; EMG: Electromyography; AR: Augmented reality; VR: Virtual reality

Through the systematic exploration presented in this paper, it is evident that both EEG and EMG technologies play pivotal roles in bridging the gap between neuroscience and robotics research. The convergence of neuroscience and robotics, underpinned by EEG and EMG (Fig. 13), offers a promising frontier for the creation of machines that are more intuitive, adaptive, and in harmony with human intentions and states.

The fusion of EEG and electromyography research with robotics offers myriad opportunities to enhance human-robot interaction, control, and navigation. These technologies have the potential to revolutionize the field by providing direct insights into users’ cognitive and muscular intentions. However, significant challenges must be addressed to fully harness the benefits of EEG and EMG in robotics.

### Challenges

The applications of EEG and EMG in robotics, notably in human-robot interactions, robot control, and navigation, represent a transformative step in leveraging biological signals for sophisticated machine control. However, it must be acknowledged that the practical implementation of these technologies is not without limitations. Issues pertaining to signal noise, user-specific variability, and potential ethical and safety considerations underscore the complex nature of this promising field of research.

#### Signal noise and variability

Both EEG and EMG signals are prone to noise and variations caused by factors such as electrode placement, user movement, and external interference. Ensuring the reliability and accuracy of signal interpretation in real-world scenarios requires robust pre-processing and signal enhancement techniques.

#### Interpretation complexity

Decoding users’ intentions from EEG and EMG signals is complex, as these signals are influenced by a multitude of cognitive and physiological processes. Developing advanced machine learning algorithms capable of accurately and quickly translating these signals into meaningful robotic actions is a significant challenge.

#### User adaptation and training

Effective utilization of EEG and EMG technologies often requires users to undergo training and adaptation periods. Ensuring that users can efficiently learn to control robotic systems and that these systems can adapt to the changing cognitive and physiological states of users is an ongoing challenge.

#### Ethical and privacy considerations

As EEG and EMG data provide intimate insights into users’ cognitive and motor functions, ethical concerns regarding data privacy, ownership, and potential misuse arise. Developing transparent and secure data-handling practices is essential for building user trust in these technologies.

Despite these challenges, the use of EEG and EMG in robotics is emerging. The prospect of improved signal processing techniques, the development of hybrid systems combining EEG and EMG, advances in user training and interface design, and the growth of fields such as neuromorphic engineering signals an exciting future.

### Opportunities

#### Enhanced human-robot interaction

EEG and EMG technologies enable robots to better understand human intentions, emotions, and motor actions. This opens doors to intuitive and natural interactions, where robots can adapt their behavior based on users’ cognitive and emotional states, leading to more empathetic and responsive human-robot interactions.

#### Precise robotic control

EEG and EMG provide fine-grained control over robotic systems. EEG-based control allows users to manipulate robots based on their cognitive intentions, whereas EMG-based control translates user muscle activations into specific robotic actions. These precise control mechanisms enable robots to perform tasks with high accuracy and agility.

#### Assistive technology and rehabilitation

EEG and EMG research can greatly benefit individuals with motor disabilities. Brain-controlled robotic prosthetics and exoskeletons powered by EMG signals can potentially restore mobility and independence compared with those with limited movement capabilities [205]. In addition, EEG-based rehabilitation systems can facilitate neural recovery after neurological injury.

#### Personalization and adaptation

EEG and EMG data provide insights into individual differences in cognitive and motor patterns. This allows the creation of personalized robotic systems that adapt to the unique physiological and cognitive characteristics of users, thereby enhancing their overall user experience and system performance.

#### Technological opportunities

Moreover, models should tolerate day-to-day physiological drift and electrode shifts with minute-scale few-shot updates and lifelong adaptation while keeping privacy and operator effort low. Fusion must be constrained by the robot’s control contracts–treat EMG as fast continuous modulation and EEG as sparse high-level gating–and realized with uncertainty-aware arbitration and predictive safety filters that preserve passivity, latency budgets, and impedance limits. Robustness and generalization call for self-supervised pretraining and digital-twin/domain-randomized augmentation that reflect neuromuscular and contact dynamics, whereas haptics/FES are co-optimized with decoders under cognitive-load estimation to improve embodiment without fatigue. Finally, progress should be measured using a two-stage benchmark coupling offline decoding with closed-loop tasking under perturbations, reporting task success, near misses, recovery time, and workload with robot operating system -compatible tooling for reproducibility.

In conclusion, the fusion of EEG and EMG with robotics not only opens up new possibilities in research and technological innovation but also promises profound societal impacts. These range from enhancing the quality of life of individuals with disabilities to revolutionizing industrial practices. As this exciting field continues to evolve, it calls for a collaborative research approach that is cognizant of the challenges but also optimistic about the opportunities that lie ahead. It is the collective responsibility of the scientific community to shepherd this potent technology toward its full potential while maintaining the highest standards of safety and ethics.

EEG and EMG research in robotics presents a wealth of opportunities for transformative advancements in human-robot interactions and control. However, these opportunities are accompanied by challenges requiring multidisciplinary collaboration to develop innovative solutions. Overcoming these challenges will pave the way for seamless integration of EEG and EMG technologies into the realm of robotics, revolutionizing the interaction and collaboration between humans and robots.

## Data Availability

All materials summarized in this article are available in the cited publications. Any illustrative figures were created by the authors from publicly available information or are original to this work.

## Abbreviations

EEG: Electroencephalography
EMG: Electromyography
sEMG: Surface electromyography
nEMG: Needle electromyography
BCI: Brain-computer interface
BMI: Brain-machine interface
HRI: Human robot interaction
SSVEP: Steady-state visually evoked potential
MI: Motor imagery
P300: P300 event-related potential
RSVP: Rapid serial visual presentation
ERP: Event-related potential
ITR: Information transfer rate
CNN: Convolutional neural network
RNN: Recurrent neural network
LSTM: Long short-term memory
TCN: Temporal convolutional network
SVM: Support vector machine
LDA: Linear discriminant analysis
KNN: K-nearest neighbor
RF: Random forest
CCA: Canonical correlation analysis
FCCA: Filter canonical correlation analysis
ICA: Independent component analysis
MAV: Mean absolute value
RMS: Root mean square
WL: Waveform length
ZC: Zero crossing
SSC: Slope sign change
MNF: Mean frequency
MDF: Median frequency
FES: Functional electrical stimulation
AR: Augmented reality
VR: Virtual reality
fNIRS: Functional near-infrared spectroscopy
BLE: Bluetooth low energy
AI: Artificial intelligence
